# Quality of Life of Patients With Chronic Venous Insufficiency of the Lower Extremities Before and After Endovascular Laser Ablation: A Prospective Pilot Study Using the Chronic Venous Insufficiency Quality of Life Questionnaire 20 (CIVIQ-20)

**DOI:** 10.7759/cureus.41854

**Published:** 2023-07-13

**Authors:** Lam Thao Cuong, Ho Tat Bang, Tran Le An, Le Thi Thien Nga, Tran Thanh Vy

**Affiliations:** 1 Department of Thoracic and Vascular Surgery, University Medical Center, Ho Chi Minh City, Ho Chi Minh, VNM; 2 Department of Cardiovascular and Thoracic Surgery, Faculty of Medicine, University of Medicine and Pharmacy at Ho Chi Minh City, Ho Chi Minh, VNM; 3 Department of Health Organization and Management, Faculty of Public Health, University of Medicine and Pharmacy at Ho Chi Minh City, Ho Chi Minh, VNM; 4 Faculty of Public Health, University of Medicine and Pharmacy at Ho Chi Minh city, Ho Chi Minh, VNM

**Keywords:** lower extremities, endovenous laser ablation, chronic venous insufficiency, civiq-20, quality of life

## Abstract

Background

Chronic venous insufficiency of the lower extremities is a condition in which blood stagnates in the venous system, causing symptoms of pain, fatigue, leg edema, and cramps. Although this disease progresses slowly and is not fatal, its symptoms lead to a decline in quality of life, affecting the patient's work. One of the current surgical treatment methods for patients with indications for surgical removal of the saphenous vein is endovenous laser ablation (EVLA). After EVLA, besides evaluating the effectiveness of surgery, and the rate of complications, one of the aspects that need attention is improving quality of life. The objective of this study was to assess the quality of life of patients with chronic venous insufficiency of the lower extremities before and after endovascular laser intervention, analyzing changes in ChronIc Venous Insufficiency Quality of Life Questionnaire 20 (CIVIQ-20) scores in each domain of quality of life.

Methodology

A total of 41 patients with chronic venous insufficiency of the lower extremities were enrolled in this study, who were classified as C2 to C6 and were treated by endovenous laser ablation. QoL was measured by using the ChronIc Venous Insufficiency Quality of Life Questionnaire 20 (CIVIQ-20) questionnaire pre- and post-operatively. Pain scores and clinical severity were also evaluated using the visual analog scale (VAS) and the venous clinical severity score (VCSS) one month after EVLA.

Results

The mean age was 52.17 ± 11.23 years. The mean duration of symptoms was 7.29 ± 3.58 years, the median laser ablation time was 40 minutes, and the mean time to return to normal activities was 5.7 ± 1.8 days. The CIVIQ-20 score at baseline was 47.8 ± 7.4 and 31.1 ± 3.9 after one-month laser ablation. The quality of life has significantly improved after intervention using laser treatment (p < 0.001). The VAS and VCSS scores after laser treatment were lower than the baseline (p < 0.001). In our study, there was a statistically significant correlation between the change of CIVIQ-20 and the change of VCSS (r = 0.63, p < 0.001).

Conclusion

CIVIQ-20 is a reliable toolkit to assess the quality of life of patients with chronic venous insufficiency of the lower extremities. The patient's quality of life improved markedly after the intervention.

## Introduction

Venous insufficiency of the lower extremities is a condition in which blood stagnates in the venous system, causing symptoms of pain, fatigue, leg edema, and cramps. Although this disease progresses slowly and is not fatal, its symptoms lead to a decline in quality of life (QoL), affecting the patient's work [1]. One of the current surgical treatment methods for patients with indications for surgical removal of the saphenous vein is endovenous laser ablation (EVLA) [2,3]. After EVLA, besides evaluating the effectiveness of surgery, and the rate of complications, one of the aspects that need attention is improving quality of life.

There are many scales of quality of life, ChronIc Venous Insufficiency Quality of Life Questionnaire 20 (CIVIQ-20) is a specialized scale for evaluating chronic venous insufficiency (CVI) of the lower extremities and is commonly used globally because of its high reliability and availability in many language versions, including Vietnamese [4]. In Vietnam, there are very few studies assessing the quality of life of patients with chronic venous insufficiency of the lower extremities using a specialized scale. Most domestic studies only assessed the overall quality of life, with no studies specifically analyzing each domain of quality of life in the CIVIQ-20. The objective of this pilot study was to assess the quality of life of patients with chronic venous insufficiency of the lower extremities before and after endovascular laser intervention, analyzing changes in CIVIQ-20 scores in each domain of quality of life.

## Materials and methods

Study settings and design

The longitudinal pilot study, pre-posterior evaluation was conducted at the University Medical Center in Ho Chi Minh City, Vietnam between January and May 2023. This study protocol and ethics were approved by the Medical Ethics Committee of the University of Medicine and Pharmacy at Ho Chi Minh City (approval number: 22904/ĐHYD). All procedures in the study were performed according to the Declaration of Helsinki.

Participants, sample size, and sampling

Our study used the formula to calculate the sample size to compare two average (before and after) pairs with µdiff = 13, 4 and σdiff = 11, 6 based on Karathanos' study [5], Z1−α/2 = 2.58 and Z1−β = 0.84. After expecting a 30% sample loss (patients lose contact after one month of intervention), we calculated the sample size as n = 30.

We interviewed 41 patients who had been diagnosed with chronic venous insufficiency of the lower extremities and indicated endovascular laser intervention. The inclusion criteria are: (1) patients diagnosed with chronic venous insufficiency of the lower extremities (clinically with symptoms such as pain, heaviness in the legs, numbness of the legs, and cramps. Clinical grade from C2 to C6. Ultrasound: there is a venous reflux current on Doppler ultrasound); (2) the patient agrees to do endovascular laser intervention at University Medical Center; and (3) the patient consented to participate in the study. The exclusion criteria were: (1) the patient was not contacted after one month of intervention; (2) the patient did not complete the pre- and post-treatment questionnaires.

Convenient sampling was used in the study to select all patients with chronic venous insufficiency of the lower extremities for endovascular laser examination and intervention. Research data collection was carried out continuously until a sufficient number of sample sizes were obtained. The investigator meets directly with the patient prior to the intervention, explaining the purpose of the study, the benefits of participating, the possible risks, and the patient's information security. Particularly emphasizing patients, the study will conduct the survey twice: before the intervention and one month after the intervention. After the patient agrees, the first questionnaire survey-the pre-intervention survey questionnaire will be taken, and the patient's appointment one month after surgery will continue to take the survey again-a set of post-intervention survey questions.

Data collections and tools

The CIVIQ-20 scale consists of 20 patient-answered, self-answered questions to assess the symptoms that occur in the legs and how much it affects the patient's quality of life (QoL). The set of questions will focus on four aspects of QoL: pain (verses 1 to 4), physical (verses 5, 6, 7, 9), social (verses 8, 10, 11), and psychological (verses 12 to 20). The set of questions will score each sentence on a five-point Likert scale: from 1 = "No impact" to 5 = "Serious impact” and will give the final score of the total score of 20 questions. To improve the CIVIQ-20 score, the QoL must be enhanced, meaning that the higher the CIVIQ-20 score, the lower the QoL [4].

The visual analog scale (VAS) is a simple scale, shaped as a horizontal or vertical line segment with a length of 100 mm and fixed at the ends from the degree 0 = "painless" to 100 = "unbearable pain". The patient will conduct a marking on a measuring 100 mm in length; the score will be calculated from the "pain-free" score to the point at which the patient marks it.

The venous clinical severity score (VCSS) scale consists of 10 questions about signs of venous pathology; each question is scored on a scale from 0 = "nothing" to 3 = "severe," and the total score will range from 0 to 30 score.

We collect baseline characteristics, risk factors, and clinical features such as symptoms, clinical-etiology-anatomy-pathophysiology (CEAP) classification, duration of disease, duration of intervention, duration of normal activities, and complications. VAS, VCSS, and CIVIQ-20 scores will be assessed and monitored twice before and after one month of intervention.

Statistical analysis

We use the paired T-test and Wilcoxon sign test to verify differences in VAS, VCSS, and CIVIQ-20 scores before and after the intervention, depending on the distribution of the data. A p-value <0.01 was considered statistically significant. We used STATA Version 16.0 (Stata Statistical Software: Release 16, StataCorp LLC, College Station, USA) to analyze and process the data.

## Results

A total of 41 patients underwent endovascular laser intervention and completed a set of pre- and post-intervention questionnaires. Socio-demographic characteristics are presented in Table 1. The mean age of study participants was 52.2 ± 11.2 years. The age group of 50 and older has a majority of 58.5%. There were 32 females, with a rate of 78.1%. The mean body mass index (BMI) was 24.1 ± 2.4 kg/m^2^, ranging from 19.2 to 31.1 kg/m^2^. About 31.7% of patients were farmers, 22 of 41 patients had other occupations (53.7%) and the rest were workers (4.9%), salespeople (7.3%), and health workers/teachers (2.4%). In the group with other occupations, there were 14 out of 41 housewives (34.1%), two entrepreneurs, two retirees, and four freelancers.

**Table 1:** Patient’s demographics (n = 41). Data are n (%), mean ± standard deviation (min–max), or median (interquartile range). BMI: body mass index.

Variable	n (%)
Age	52.2 ± 11.2 (28–71)
Age group	
<50	17 (41.5)
≥50	24 (58.5)
Sex	
Male	9 (21.9)
Female	32 (78.1)
BMI	24.1 ± 2.4 (19.2–31.1)
Job	
Farmer	13 (31.7)
Worker	2 (4.9)
Salesperson	3 (7.3)
Health workers/teachers	1 (2.4)
Different	22 (53.7)

The patient's risk factors are shown in Table 2. There were 25 (78.1%) females who have been pregnant two or more times, 6 (18.8%) have been pregnant a single time, and the remaining one has never been pregnant. There were 32 patients (82.9%) who had the activity of standing or sitting for eight hours or more in a day, and the remaining 17.1% did not. There were 51.2% of patients who had a family history of chronic venous insufficiency (CVI). 

**Table 2:** The patients’ risk factors (n = 41).

Variable	n (%)
Number of pregnancies (n = 32)	
Never before	1 (3.1)
One time	6 (18.8)
≥2 Times	25 (78.1)
Standing/sitting ≥8 hours/day	
Yes	34 (82.9)
No	7 (17.1)
Family history of chronic venous insufficiency	
Yes	21 (51.2)
No	20 (48.8)
Regular physical activity	
Yes	17 (41.4)
No	24 (58.6)
Overweight	
Yes	30 (73.2)
No	11 (26.8)

The duration of symptoms was 7.29 ± 3.58 years. Of the 41 study participants, 29 (70.7%) received endovascular laser intervention on both legs, 17.1% received the intervention on the right leg, and 12.2% on the left leg. The majority of symptoms of chronic venous insufficiency of the lower extremities were present in patients with severe lower leg pain (100%), lower leg pain (95.1%), cramps (92.7%), and burning in the legs (85.4%) (Table 3).

Because the study on patients indicated endovascular laser intervention, patients with chronic venous insufficiency of the lower extremities participating in the study must be between stages C2 and C6. Before the intervention, 63.4% of patients are classified as C2, 11 (26.8%) people are in stage C3, and stage 4 (9.8%) people who progress through stage C4. After one month of intervention, 61% of patients were in stage C0, 29.3% in stage C1, 7.3% in stage C2, and one patient was in stage C3.

**Table 3:** Clinical, subclinical features of the patients. Data are n (%), mean ± standard deviation (min–max), or median (interquartile range). CEAP: clinical-etiology-anatomy-pathophysiology.

Duration of symptoms (year)	7.29 ± 3.58 (2–17)
Extremities involved	
Left leg	5 (12.2)
Right leg	7 (17.1)
Both legs	29 (70.7)
Symptoms	
Heaviness	41 (100)
Pain	39 (95.1)
Edema	15 (36.6)
Cramp	38 (92.7)
Burning	35 (85.4)
Itching, numbness, stinging	18 (43.9)
Change skin color	4 (9.8)
CEAP classification before intervention	
C2	26 (63.4)
C3	11 (26.8)
C4	4 (9.8)
CEAP classification after intervention	
C0	25 (61)
C1	12 (29.3)
C2	3 (7.3)
C3	1 (2.4)
Time of duration of laser treatment (minute)	40 (30–45)
Time to return to normal activities (day)	5.7 ± 1.8 (2–10)

**Figure 1:**
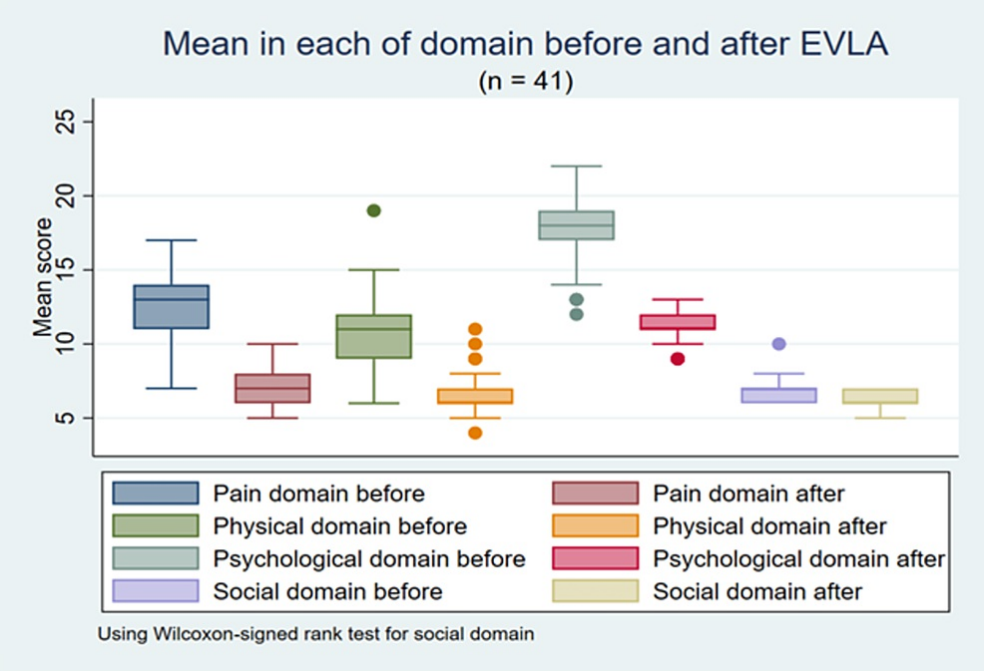
CIVIQ-20 scores (four domains) are recorded preoperatively and at the one-month follow-up. CIVIQ-20: Chronic Venous Insufficiency Quality of Life Questionnaire 20, EVLA: endovenous laser ablation.

Before the intervention, the average VAS score was 6.2 ± 0.7 and after the intervention was 1.9 ± 0.6, this difference was statistically significant (p < 0.001). A statistically significant decrease in VCSS scores was statistically significant after one month of intervention (down 3.2 scores, p < 0.001) (Table 4). Statistically significant reductions in CIVIQ-20 scores were statistically significant across all areas of quality of life (p < 0.001). There were statistically significant differences in each domain of quality of life (p < 0.001) (Figure 1).

**Table 4:** VAS, VCSS, and CIVIQ-20 scores are recorded preoperatively and at the one-month follow-up. Data are mean ± standard deviation, or median (interquartile range). Use the Wilcoxon-sign ranked test for the social domain in CIVIQ-20. VAS: visual analog scale, VCSS: venous clinical severity score, CIVIQ-20: Chronic Venous Insufficiency Quality of Life Questionnaire 20.

	Pre-intervention	Post-intervention	p-value
VAS	6.2 ± 0.7	1.9 ± 0.6	<0.001
VCSS	5.9 ± 2.4	2.7 ± 1.6	<0.001
CIVIQ-20	47.8 ± 7.4	31.1 ± 3.9	<0.001
Pain	12.5 ± 2.4	7.2 ± 1.3	<0.001
Physical	10.6 ± 2.7	6.5 ± 1.4	<0.001
Psychological	17.7 ± 2.5	11.1 ± 1.1	<0.001
Social	7 (6–7)	6 (6–7)	<0.001

## Discussion

First of all, regarding the socio-demographic characteristics of chronic venous insufficiency (CVI) patients, our results are similar to those of studies around the world. The mean age was 52.17 ± 11.23 years, the youngest patient was 28 years old and the oldest was 71 years, the age group 50 and above accounted for the highest rate of 58.54%. Similarly, the results of Hang et al. recorded a mean age of 55 years, and the group of 50 years and above accounted for 52.7% [6]. Previous studies have also shown similar age results, with the mean age of CVI patients being 53.5 ± 12.4 years [7]; 58.85 ± 11.63 years old [3]. The study included 41 patients, of whom women accounted for 78% and had rates 3.5 times higher than men. Research by Özkan et al. shows that the proportion of women is 63% and men 27% [8]. Other studies have shown similar results, with Kempeneers et al. 66% female and 34% male [9]. The reason for the difference in the higher incidence of the disease in women than men is the variability of female sex hormones during pregnancy [10]. Our study was dominated by 80.5% standing/sitting occupations (34.2% housewives, 31.7% farmers, 7.3% salespeople, 4.9% workers, and 2.4% health workers/teachers). Our study was interested in these occupational groups because they were associated with prolonged standing and sitting in each occupational group. Excessive standing position in one place is a risk factor for CVI, the mechanism of the disease is to increase venous pressure and increase blood flow stagnation in the veins of the lower extremities. Previous studies have shown consistent results, with a 79.4% proportion of CVI in the standing/sitting occupation group [11]. A study by Ngoc et al. conducted at Bach Mai Hospital in Vietnam showed that the majority of patients have a farming occupation rate of 41.5%, and a trade rate of 19.5% [12].

Based on previous studies, the incidence of CVI is higher in women who have been pregnant compared to women who have never been pregnant, and women who have multiple pregnancies contribute to a higher incidence than usual [13]. The majority of female patients were pregnant two times, with a rate of 78.1%, and 3.1% had never been pregnant once. The results were similar to previous studies. According to Anh, the number of births occurring two or more times was 98.1% [14]. The incidence of CVI was increased in the group with prolonged standing activity and prolonged sitting in one place for more than eight hours per day [14]. Prolonged standing and sitting are thought to lead to increased venous pressure due to the stagnation of blood in the veins, which in the long run will cause chronic venous insufficiency of the lower extremities. In our study, the majority of patients who had the factor of standing/sitting for more than eight hours a day had a rate of 82.9%. The results are similar to previous studies; according to Hung and colleagues, the rate of standing/sitting for a long time is 97.5% [15], according to Trieu et al., this rate is 97.1% [16] and according to Hang et al., it is 86% [6]. According to Thuan et al., the proportion of people with CVI who are physically active is 35.3% [11]. In our study, the proportion of people with chronic venous insufficiency of the lower extremities who were physically active was 41.4%. The reason for this difference may be that our study has an average age of working age, and it may be due to discomfort that symptoms of CVI should limit physical activity.

In our study, the median time to detection of chronic venous insufficiency of the lower extremities to the decision to undergo endovascular laser intervention was 7.29 ± 3.58 years; the lowest was two years and the highest was 17 years. Our results were lower than those of Ozkan et al., where the duration of symptoms to the intervention had a median of 10 years [8]. However, our results are similar to those of Trieu and colleagues, who showed that the average detection time was 6.1 ± 1.8 years in the counseled group and 8.5 ± 3.6 years in the non-counseled group [16]. The reason for this difference may be that the distribution of sample sizes is uneven between studies, different background characteristics in each study participant in different countries, and possibly depending on the patient's CEAP classification. Our study was dominated by patients in groups C2 and C3, so the detection time to intervention may be longer than studies with patients in the higher CEAP clinical classification.

Symptoms can be seen as the reason or complaint of the patient upon admission. In our study, the symptoms that appeared in patients were 100% higher leg heaviness, 95.1% leg pain, 92.7% cramps, 85.4% burning in the legs, 43.9% stinging numbness, 36.6% swelling of the legs, and skin color change, with the lowest rate of 9.8%. In a study evaluating two endovascular treatments, in the group that received endovascular lasers, the most common symptoms were pain, severe calf pressure at 95.7%, cramps at 87.1%, burning legs at 72.9%, leg edema at 50.7%, and itching at 39.3% [14]. The results of our study are almost identical to the author's results on symptoms of severe leg pain, cramps, burning legs, and itchy feet. It is possible that the majority of patients in the study group have a clinical grade of C2. At this stage, the patients do not develop symptoms of leg edema, so the symptoms of leg swelling in our study are lower than the authors.

Because the study involved patients treated by endovenous laser ablation (EVLA), the patients with chronic venous insufficiency of the lower extremities in our study ranged from C2 to C6. Of the total 41 study participants, the majority of patients in stage C2 according to the CEAP clinical classification had a rate of 63.4%, stage C3 26.8%, stage C4 9.8%, and no patients in stages C5 or C6. According to Hung et al., the results showed that the proportion of people with CVI in stage C2 was 58.5%, C3 was 26.9%, C4 was 13.4%, and C5 was 0.8% [15]. Our results are closely similar to previous studies, the reason for this difference is that the sample size in our study was smaller than in previous studies, so no patients in stages C5 and C6 were recorded. A study by Kempeneers et al. found that the proportion of people with CVI after EVLA in stage C0 was 80%, C1 was 6%, C2 was 10%, C3 was 3%, and C4 was 1% [9]. In our study, the proportion of patients in stage C0 was 61%, stage C1 was 29.3%, stage C2 was 7.3%, stage C3 was 2.4%, and no patients were recorded in stage C4 or higher. Clinical improvement by CEAP classification indicates the effectiveness of EVLA for CVI patients.

In our study, 29 patients were EVLA on both legs at a rate of 70.7%, and seven people received the intervention on the right leg at a rate of 17.1% and five people on the left leg at a rate of 12.2%. The duration of the intervention is the period of time between the start and end of laser intervention. In our study, the duration of endovascular laser intervention had a median of 40 minutes, the lower quartile was 30 minutes, and the upper quartile was 45 minutes. Our results are consistent with the study of Duc et al., which showed that the average treatment time was 43.77 ± 11.96 minutes [2]. In addition, our results were lower than those of Mekako and colleagues with a median intervention duration of 69 minutes (IQR: 60-80 minutes) [17]. The reason for this difference may be that the endovascular laser technique uses different wavelengths, our study site uses a wavelength of 1470 nm, and Mekako uses a wavelength of 810 nm. In our study, the median time to return to normal activities after endovascular laser intervention was 5.7 ± 1.8 days, with the lowest being two days and the highest being 10 days. The results are consistent with the study of Shepherd and colleagues, with results showing that the time to return to normal activity in the group receiving endovascular laser intervention had a median of five days (0-11 days) [18]. However, our results were higher than the study of Duc and colleagues with a return time of 2.13 ± 0.59 days [2]. The reason for this difference may be that patients in our study are older, the proportion of women is 3.5 times higher than that of men, so patients may have psychological fear, limited movement, and early activities for fear of affecting the incision (Table 5).

**Table 5:** Some similar studies on the quality of life of patients with CVI. RFA: radiofrequency ablation, CVI: chronic venous insufficiency, EVLA: endovenous laser ablation.

Authors	Study location	Study design	Sample size
Duc et al. [2]	Vietnam	A prospective follow-up	Sixty-one patients
Phuc et al. [3]	Vietnam	A before and after study	Four hundred ninety-eight patients
Hang et al. [6]	Vietnam	A prospective follow-up	Nine thousand two hundred thirty patients
Phuong [7]	Vietnam	A prospective follow-up	Seventy-five patients
Özkan et al. [8]	Turkey	A prospective follow-up	Thirty-eight patients
Kempeneers et al. [9]	Belgium	A prospective RCT	One hundred forty-two patients for EVLA and 138 patients for RFA
Anh [14]	Vietnam	A prospective follow-up	One hundred forty patients
Mekako et al. [17]	UK	A prospective follow-up	Sixty-seven patients
Shepherd et al. [18]	UK	A prospective RCT	Thirty-five patients for EVLA and 46 patients for RFA
Chen et al. [19]	China	A prospective follow-up	Thirty-one patients
Vourliotakis et al. [20]	Greece	A prospective follow-up	One hundred seventy patients
Karathanos et al. [5]	Greece	Cohort study	One hundred fifty-three patients

The mean VAS score before the intervention was 6.2 ± 0.7, dropping to 1.9 ± 0.6 points after the intervention, and this difference was statistically significant (p < 0.001). Our results are closely consistent with those of Chen et al., the mean VAS score after one month of intervention was 1.32 ± 0.64, a significant decrease compared to one week before the intervention of 4.56 ± 1.89 points, and this difference was statistically significant (p < 0.05) [19]. In our study, we had a higher pre-intervention VAS score than the author's study because we assessed the pre-intervention VAS score at the time the patient was hospitalized and waiting for intervention, while the study by Chen et al. recorded VAS scores of one week before the intervention. A follow-up study in Greece of 153 patients aimed to assess VAS scores pre-intervention, one week, one month, and one year after endovascular intervention [5]. The results showed that in the group receiving endovascular laser intervention with jacket-tip fiber (EVLA-J), VAS scores before the intervention and after one month of intervention were 5.3 ± 1.6 and 0.9 ± 1.4, respectively. Because the study wanted to compare between groups, no matching test was performed to compare VAS scores before and after.

According to Karathanos et al., the average VCSS score before the intervention was 6.6 ± 2.8, and after one month was 4.9 ± 2.2, specifically the average VCSS score decreased by 1.7 points after one month of endovascular laser intervention [5]. According to research by Ozkan et al., pre-intervention VCSS scores had a median of 5 (1-21) and post-intervention 3 (0-12), VCSS scores decreased significantly after endovascular laser intervention, which was statistically significant (p < 0.001) [8]. Our study used a paired T-test to evaluate VCSS score changes before and after one month of endovascular laser intervention. Our results showed that VCSS scores decreased from 5.9 ± 2.4 points to 2.7 ± 1.6 points and that there was a significant difference between statistically significant pre- and post-intervention VCSS scores (p < 0.001). According to Anh et al., VCSS scores before and after one month of endovascular laser performance of patients were 5.0 ± 2.0 and 3.4 ± 1.9, respectively, a statistically significant decrease (p < 0.001) [14]. A study by Phuc et al. also showed similar results to our study, the pre-intervention VCSS score of 4.6 ± 1.8 decreased to 1.1 ± 0.7 after the intervention, and the difference in VCSS scores before and after was statistically significant (p < 0.001) [3].

Our study found that the mean CIVIQ-20 score before the intervention was 47.8 ± 7.4, and after one month of intervention, it dropped to 31.1 ± 3.9 points. This difference was statistically significant (p < 0.001). In terms of each field, the psychological field changed the most with pre-intervention scores of 17.7 ± 2.5, a decrease of 6.5 scores one month after the intervention, and this difference was statistically significant (p < 0.001). The pain area saw a significant decrease in mean score after the intervention (12.5 ± 2.4 and 7.2 ± 1.3, p < 0.001), and scores in the physical field also decreased significantly after the intervention (10.6 ± 2.7 and 6.5 ± 1.4, p < 0.001). In the domain of social issues, the average score difference between pre- and post-intervention has a skewed distribution, so we used the Wilcoxon signed rank test to assess the change before and after. The social field score before the intervention had a median of seven points, and after one month the median was six points, the difference was statistically significant (p < 0.001). According to research by Vourliotakis et al., the results showed that the mean CIVIQ-20 score before the intervention was 77 ± 3.9, a significant decrease of 36.3 ± 3 points after one month of intervention, and this difference was statistically significant (p = 0.001) [20]. Based on these results, the study also acknowledged that patients' quality of life was low at the time before the intervention, and after the intervention, the quality of life was increased. The study suggested that significantly reduced pain field scores resulted in a significant difference in CIVIQ-20 scores, with pre- and post-intervention pain field scores of 17.4 ± 1.3 and 6.7 ± 1.2, respectively, a statistically significant difference (p = 0.001). In addition, the authors recorded mean scores before and after the intervention in the physical (28.6 ± 2.8 and 14 ± 1.9, p = 0.001), psychological (18.5 ± 2 and 9 ± 1.5, p = 0.001), and social (12.4 ± 1.3 and 6.6 ± 1.5, p = 0.001). The study concluded that patients' quality of life improved very well after endovascular laser intervention, and the most significant change was in the area of pain. The author's results were higher than in our study, in which we had lower pre-intervention CIVIQ-20 scores, possibly because the patients in our study were mostly clinically mild and had a lower time to detect the disease than the author. One study assessed the quality of life of patients with chronic venous insufficiency of the lower extremities in three groups that received different interventions and compared the differences between the three groups [5]. The study results showed that QoL had significant changes after one month of endovascular laser intervention, with mean CIVIQ-20 scores before and after being 38.1 ± 11.6 and 24.7 ± 9.1, respectively. The study found that in areas of quality of life, the area of pain changed significantly after the intervention. Mean score before and after in the pain domain (9 ± 3.1 and 4.9 ± 3), physical domain (7.2 ± 2.7 and 4.1 ± 0.4), psychological domain (15.9 ± 5.3 and 11.5 ± 2), and social domain (5.6 ± 2.6 and 3.4 ± 0.8). The results show that chronic venous insufficiency of the lower extremities affects all areas of the patient's life, reducing the quality of life and after the intervention, the quality of life is significantly improved in all areas of life. The author's results were lower than our study, possibly because patient characteristics differed in each study and the majority of patients in the author's study were at a milder clinical stage than us. Domestic studies conducted in patients with chronic venous insufficiency of the lower extremities have mostly been a general assessment of changes in quality of life before and after the intervention, not area-specific changes in the CIVIQ-20 questionnaire. Our results have CIVIQ-20 before and after scores similar to Anh's study, with CIVIQ-20 scores decreased by 20 points after one month of endovascular laser intervention, CIVIQ-20 before and after scores of 45.6 ± 7.2 and 25.6 ± 6.8 (p < 0.001) [14]. The author also stated that the patient's quality of life improved very well after endovascular laser intervention and according to Phuc et al., the results showed that the CIVIQ-20 score decreased statistically significantly after the intervention, the average CIVIQ-20 score before and after the intervention was 54.0 ± 7.8 and 27.8 ± 3.5 (p < 0.001) [3].

Regarding VCSS scores, we noted a strong positive correlation between CIVIQ-20 scores before and after and VCSS scores before and after, which was statistically significant (r = 0.63, p < 0.001).

This is a before-and-after study, assessing changes in the quality of life of patients with chronic venous insufficiency of the lower extremities specific to each area of life and using the CIVIQ-20 scale to assess the quality of life. Our study had a limited sample size and was conducted over a short period, so it does not generalize the change in the quality of life of patients with chronic venous insufficiency of the lower extremities before and after endovascular laser intervention in the population. Due to study time constraints, as well as domestic and foreign studies that only broadly assess changes in patients' pre- and post-intervention quality of life, we cannot discuss any changes in CIVIQ-20 scores in each specific area.

## Conclusions

CIVIQ-20 is a reliable toolkit to assess the quality of life of patients with chronic venous insufficiency of the lower extremities. CIVIQ-20 scores decreased significantly after one month of intervention, especially in areas where pain improved better than in other areas. 

## References

[1] Youn YJ, Lee J (2019). Chronic venous insufficiency and varicose veins of the lower extremities. Korean J Intern Med.

[2] Duc HK, Thanh DH, Hai PT, Thinh CV, Minh NC, Tan V (2014). Varicose vein treatment with endovenous laser therapy: the outcome after 2 years follow-up. Vietnam J Cardiovasc Thorac Surg.

[3] Phuc VM, Quan HV, Son NV, Hung TD (2022). Experience with 1470 nm laser ablation treatment for 689 limbs with lower extremity superficial venous insufficiency. Vietnam Med J.

[4] Launois R (2015). A quality of life tool kit in chronic venous disorders. Phlebolymphology.

[5] Karathanos C, Spanos K, Batzalexis K, Nana P, Kouvelos G, Rousas N, Giannoukas AD (2021). Prospective comparative study of different endovenous thermal ablation systems for treatment of great saphenous vein reflux. J Vasc Surg.

[6] Hang LTN, Dinh LQ, Luan TMB (2018). Treatment outcomes of chronic venous insufficiency for 9230 patients at University Medical Center, Ho Chi Minh city. Vietnam J Cardiovasc Thorac Surg.

[7] Phuong DD (2018). Evaluation of clinical efficacy on a VDS scale in the treatment of venous insufficiency of the lower extremities with endovascular lasers. Master of Surgery Thesis. Master of Surgery Thesis, University of Medicine and Pharmacy at Ho Chi Minh City.

[8] Özkan U, Saritüürk Ç (2012). Early clinical improvement in chronic venous insufficiency symptoms after laser ablation of saphenous veins. Diagn Interv Radiol.

[9] Kempeneers AC, Bechter-Hugl B, Thomis S, van den Bussche D, Vuylsteke ME, Vuylsteke MM (2022). A prospective multicenter randomized clinical trial comparing endovenous laser ablation, using a 1470 nm diode laser in combination with a Tulip-Tip™ fiber versus radiofrequency (Closure FAST™ VNUS®), in the treatment of primary varicose veins. Int Angiol.

[10] Stansby G (2000). Women, pregnancy, and varicose veins. Lancet.

[11] Thuan NTT, Nam NH (2020). Quality of life and its associated factors among patients with chronic venous insufficiency. Vietnam J Cardiovasc Thorac Surg.

[12] Ngoc NV, Thong PM, Nguyet LM, Tuan TA (2020). Study the imaging characteristics of Doppler ultrasound before and after the laser incineration of the great saphenous venous insufficiency at Bach Mai Hospital. Vietnam J Radiol Nucl Med.

[13] Dindelli M, Parazzini F, Basellini A, Rabaiotti E, Corsi G, Ferrari A (1993). Risk factors for varicose disease before and during pregnancy. Angiology.

[14] Anh NT (2017). Study of the Clinical, and Subclinical, Outcomes of Treatment of Chronic Lower Extremity Venous Insufficiency by Sclerotherapy and Intravenous Laser Method. National Geriatric Hospital.

[15] Hung TD, Thu PVH, Hai NT, Phuc VM (2018). Clinical characteristics and results of treatment of patients with superficial venous insufficiency of the lower extremities by endovascular laser method. Vietnam J Cardiovasc Thorac Surg.

[16] Trieu NB, Ha NT, Phuc VM, Hung TD (2018). Clinical characteristics and results of endovenous laser treatment for varicose veins. J Nurs Sci.

[17] Mekako A, Hatfield J, Bryce J, Heng M, Lee D, McCollum P, Chetter I (2006). Combined endovenous laser therapy and ambulatory phlebectomy: refinement of a new technique. Eur J Vasc Endovasc Surg.

[18] Shepherd AC, Gohel MS, Lim CS, Hamish M, Davies AH (2010). Pain following 980-nm endovenous laser ablation and segmental radiofrequency ablation for varicose veins: a prospective observational study. Vasc Endovasc Surg.

[19] Chen J, Xie H, Deng H (2013). Endovenous laser ablation of great saphenous vein with ultrasound-guided perivenous tumescence: early and midterm results. Chin Med J.

[20] Vourliotakis G, Sahsamanis G, Evagelidis P, Aivatidi C (2018). Endovascular laser treatment of incompetent saphenous veins using the 1470 nm diode laser and radial fiber. Ann Med Surg.

